# Evaluation of Combined Financial Incentives and Deposit Contract Intervention for Smoking Cessation: A Randomized Controlled Trial

**DOI:** 10.1155/2021/6612505

**Published:** 2021-03-22

**Authors:** Daren R. Anderson, Samantha Horn, Dean Karlan, Amanda E. Kowalski, Jody L. Sindelar, Jonathan Zinman

**Affiliations:** ^1^Department of Medicine, Yale School of Medicine, Yale University, New Haven, CT, USA; ^2^Department of Social and Decision Sciences, Carnegie Mellon University, Pittsburgh, PA, USA; ^3^Kellogg School of Management, Northwestern University, Evanston, IL, USA; ^4^Department of Economics, University of Michigan, Ann Arbor, MI, USA; ^5^Department of Health Policy and Management, Yale School of Public Health, Yale University, New Haven, CT, USA; ^6^Department of Economics, Dartmouth College, Hanover, NH, USA

## Abstract

**Introduction:**

We evaluate whether a combination of financial incentives and deposit contracts improves cessation rates among low- to moderate-income smokers.

**Methods:**

We randomly assigned 311 smokers covered by Medicaid at 12 health clinics in Connecticut to usual care or one of the three treatment arms. Each treatment arm received financial incentives for two months and either (i) nothing further (“incentives only”), (ii) the option to start a deposit contract with incentive earnings after the incentives ended (“commitment”), or (iii) the option to precommit any earned incentives into a deposit contract starting after the incentives ended (“precommitment”). Smoking cessation was confirmed biochemically at two, six, and twelve months.

**Results:**

At two, six, and twelve months after baseline, our estimated treatment effects on cessation are positive but imprecise, with confidence intervals containing effect sizes estimated by prior studies of financial incentives alone and deposit contracts alone. At two months, the odds ratio for quitting was 1.4 in the incentive-only condition (95% CI: 0.5 to 3.5), 2.0 for incentives followed by commitment (95% CI: 0.6 to 6.1), and 1.9 for incentives and precommitment (95% CI: 0.7 to 5.3).

**Conclusions:**

A combined incentive and deposit contract program for Medicaid enrollees, with incentives offering up to $300 for smoking cessation and use of support services, produced a positive but imprecisely estimated effect on biochemically verified cessation relative to usual care and with no detectable difference in cessation rates between the different treatment arms.

## 1. Introduction

Smoking rates have declined dramatically in the United States but remain high in the Medicaid population [1, 2]. Medicaid enrollees are less likely to use formal cessation services in quit attempts and are less successful in quitting when they try (HHS, 2018; [1, 3–6]). Consequently, this population continues to suffer from high rates of chronic smoking-related diseases [7] For example, nationally representative data on 18-65-year-old respondents to the 2015 National Health Interview Survey shows chronic obstructive pulmonary disease (COPD) rates of 6.2% among Medicaid enrollees compared to 1.2% for privately insured respondents.

We developed and tested a novel approach to smoking cessation incentives, in an attempt to create a cost-effective intervention for Medicaid populations. Specifically, to reinforce standard cessation efforts, we combined incentives with deposit contracts that could be funded with earned incentives.

Financial incentives have been shown to improve smoking cessation rates in many populations [8]. However, there have been mixed results on the impact of incentives for Medicaid populations [9, 10]. Further, although some studies find that financial rewards improve smoking cessation rates when measured at twelve months [11–13], many others find that short-term improvements in cessation rates dissipate over time [14].

Deposit contracts, whereby participants forfeit their own money if a cessation target is not met, are effective on average. However, they exhibit low take-up rates as people seem reluctant to put their own money on the line [11, 15–17]. Combining financial incentives for cessation with the opportunity to commit incentive earnings to a deposit contract could make incentive-induced cessation more persistent, at no additional program costs, by increasing participants' willingness to take up deposit contracts.

To test the efficacy of our combined financial incentives and deposit contract intervention, we randomly assigned 311 smokers covered by Medicaid to usual care or one of the three treatment arms receiving financial incentives for two months and (i) nothing further (“incentives only”), (ii) the option to start to a deposit contract with any incentives earned, offered at the end of the incentive program (“commitment”), or (iii) the option to precommit any incentives earned to a deposit contract starting after the incentives ended, offered at the beginning of the incentive program (“precommitment”).

Our hypothesis was that financial incentives would increase cessation rates for all treatment groups relative to usual care in the short term, i.e., when measured at two months. Drawing on the deposit contract literature (for an overview, see [18]), we also predicted that participants who were offered deposit contracts would be more likely to maintain cessation in the long term, i.e., when measured at six and twelve months, because their earnings would be at risk and thus they would be less likely to revert to smoking. Finally, we anticipated that some participants may not want to sign a deposit contract when the money is “hot,” i.e., just earned, because at that moment they may be overconfident on their ability to not revert or may also have immediate ideas on how to spend the cash. Thus, our third hypothesis was that deposit contract take-up would be higher in the precommitment arm than in the commitment arm.

## 2. Methods

### 2.1. Study Sample

In November 2015, we started enrolling Medicaid participants from a large statewide Federally Qualified Health Center (FQHC) in Connecticut in a smoking cessation program. Eligibility criteria were being a daily smoker over 18 years old, wanting to quit smoking, and having a clinic visit in the past two years. Our initial aim, based on power calculations, was to recruit 1500 participants over two years. The target sample size was chosen based on power calculations to detect, with 80% power and a 5% significance threshold: (1) a treatment effect of 4 percentage points for the incentive-only arm compared to a control group mean cessation rate of 3 percent, (2) a treatment effect of the pooled deposit contract arms of 4 percentage points for the incentive arm compared to a control group cessation rate, (3) a 7 percentage point difference in treatment effect between the incentive-only and pooled deposit contract arms assuming a mean cessation rate of 7 percent in the incentive-only arm, and (4) a 9 percentage point increase in deposit contract take-up in the precommitment arm compared to the commitment arm assuming take-up of 10 percent in the commitment arm.

However, staffing challenges forced us to scale back recruitment. Specifically, the staff allocated for recruitment by clinics did not have enough time to meet recruitment targets and manage their other clinic-based duties. We lacked the resources required to hire dedicated recruitment staff and consequently ended up enrolling only 311 participants between November 2015 and October 2016.

At enrollment, participants completed a short baseline survey with questions about demographics and smoking-related behaviors. Specifically, we asked about marital status, the highest level of education completed, the number of individuals living in the household, household income, pregnancy status, and the number of hours with Internet access a week. The baseline survey also contained questions for the Fagerstrom index as well as questions about the participant's smoking history and previous quit attempts. The study was approved by the Institutional Review Board (IRB) of Community Health Center, Inc.

### 2.2. Intervention

The trial was as a multiarm parallel-group study with simple randomization and no blinding. We randomly assigned the 311 study participants, at the participant level, with a 1 : 1 : 0.5 : 0.5 split, to either usual care (*N* = 103) or one of the three treatment arms with the opportunity to earn financial incentives for two months and (i) nothing further (“incentives only,” *N* = 107), (ii) the option to start a deposit contract after the incentives ended (“commitment,” *N* = 42), or (iii) the option to precommit incentive earnings into a deposit contract starting after the incentives ended (“precommitment,” *N* = 59). Participants were randomly assigned to a group via online software designed for the implementation of randomized controlled trials. Clinic staff inputted a new participant's study identification number into the software application to obtain the participant's treatment assignment.

All study participants were encouraged to use the clinic's usual care cessation support services, including individual counselling, group counseling and nicotine replacement therapy, and the state's quitline. Clinic staff informed participants about available services in-person after enrollment and gave participants a small brochure with the same information.

Participants in the incentive-only group were offered usual care, plus $200 for biochemically verified smoking cessation measured at two months and up to $100 for cessation support activities during the first two months, including group and individual counselling, for a total possible reward of $300. Rewards were paid at the conclusion of the first two months, via a gift card redeemable at a large supermarket chain.

Participants in the commitment and the precommitment were offered deposit contracts lasting for four months after the incentive period, in addition to the same financial incentives and usual care offered to the incentive-only group. In the commitment group, after the incentive earning period, participants who were verified as having quit smoking were asked after the incentive period if they wanted to transfer all or part of their earnings into a deposit contract. Clinic staff helped interested participants through the deposit contract setup process, usually during the participant's clinic visit to verify cessation. In the precommitment group, participants had the option at baseline to automatically transfer all or part of any future earned incentive into a deposit contract. Clinic staff helped participants who elected this option set up their deposit contract during study enrollment.

During study enrollment, all participants assigned to treatment arms were registered for a study-specific portal on a website that provides goal monitoring and online deposit contracts (https://stickK.com). The portal allowed all participants to connect online with anyone who wanted to support their cessation efforts, log journal entries about their cessation progress, and sign up for usual care services. All deposit contracts were Internet-based and implemented via the same web portal. During enrollment, clinic staff provided participants with information about how to use the portal. An email address was required to sign up to the portal, and clinic staff offered participants without an email address help creating one during enrollment. Regular Internet access was not an eligibility criterion.

### 2.3. Measures

The primary outcomes are biochemically verified smoking cessation at two, six, and twelve months after enrollment. Clinic staff contacted participants by phone and asked them to make an appointment at the clinic to verify their smoking cessation status. Appointments had to be made within a two-week window of each time milestone. Only participants who reported cessation on the phone were asked to validate it with a CO breathalyzer and urine cotinine test. We defined cessation as having a CO reading below 8 ppm and a urine cotinine level below or equal to 20 ng/mL. Passing the CO test is not an outcome itself but was instead used as a filter to reduce the number of participants requiring a urine cotinine test. Any participant who did not provide biochemical verification in the form of a urine cotinine test was recorded as still smoking. Our secondary outcomes are the number of cessation support prescriptions written and the number of counseling sessions attended.

### 2.4. Statistical Analysis

All analyses compare outcomes in the usual care group to outcomes in each treatment group and to outcomes for any treatment group. Our primary analyses use multivariate logistic regressions to compare the likelihood of biochemically confirmed cessation at two, six, and twelve months. We use ordinary least squares (OLS) to compare total counseling sessions attended and total support prescriptions written across groups. All analyses are on an intent-to-treat basis and estimated with and without demographic controls (including age as a continuous measure, binary indicators for sex and high school as the highest education level, and several indicators for income bins). Analyses were conducted in 2019 using Stata version 15. We preregistered the study with ClinicalTrials.gov (study identifier: NCT02596061).

## 3. Results

All study participants (*n* = 311) had Medicaid insurance. The study group is 55% female, 45% White, and 30% Hispanic, with a mean age of 44 years (SD = 11 years) (Table 1). Seventy-two percent of participants were not college-educated, and 56% reported an annual household income of less than $10,000. Around 3% of participants smoked over 20 cigarettes a day, and according to the Fagerstrom test for nicotine dependence, 15% of participants exhibit high dependence and 32% exhibit moderate dependence. All baseline characteristics are balanced across the control group and treatment groups with the exception of a small difference in age. We run our analyses with and without baseline demographic characteristics including age for robustness.

Table 2 shows that there are no statistically significant differences in quit rates when comparing each of the treatment groups against usual care. At two months, focusing on the specification without demographic controls, the odds ratio for quitting is 1.4 in the incentive-only condition (95% CI: 0.5 to 3.5), 2.0 for incentives with commitment (95% CI: 0.6 to 6.1), and 1.9 for incentives with precommitment (95% CI: 0.7 to 5.3). Pooling treatments to increase statistical power for estimating the effect financial incentives on cessation at two months, the odds ratio is 1.6 (95% CI: 0.7 to 3.7). Nor is there any statistically significant treatment effect at either six or twelve months. At twelve months, only three participants were measured as nonsmokers (0.6% of the sample compared to 4.2% at six months and 10.6% at two months), and so there is no maximum likelihood estimate using logistic regression to report.

Our estimates for all time periods are imprecise in that they include a wide range of possible effect sizes. A recent meta-analysis of 30 studies comparing incentives (including deposit contracts) for smoking cessation against no incentives finds an odds ratio of 1.49 (CI: 1.28-1.73) at the longest follow-up (typically 6 months). Our confidence intervals include this point estimate and those from other meta-analyses of the literature [8, 19].

Table 2 also shows that there is no statistically significant increase in the utilization of smoking support services across groups. Specifically, we find no statistically significant difference in the number counselling sessions attended or cessation support prescriptions given. However, we do find high demand for a deposit contract when combined with financial incentives: a 44% take-up rate in the precommitment group (26/59), as compared to, e.g., 11% in Giné et al. [15] and 13.7% in Halpern et al. [11]. Conversely, none of the six participants in the commitment group eligible for a deposit contract at two months opted to start a deposit contract.

## 4. Discussion

We tested a novel combination of incentives and deposit contracts among clinic-based Medicaid participants who indicated interest in smoking cessation. The relatively high take-up of commitment contracts in the precommitment arm suggests that creative deposit contracts are feasible additions to financial incentive programs—the take-up rate was higher than that seen in other studies offering deposit contracts for smoking cessation without precommitment and higher than that in our commitment arm in this study. But the incentives did not increase cessation enough to permit identification of the ultimate effect of precommitment on cessation rates. The precommitted deposit contracts ended up lacking commitment value for continued smoking cessation during the two-month to six-month period for smoking cession, because the financial incentives did not induce cessation during the initial two-month period and hence most precommitted contracts were not funded.

Our study has several limitations. First and foremost, our estimates are not precise enough to make strong inferences about the efficacy of financial incentives and deposit contracts within the target population. The point estimates for the cessation rates are in line with those observed in other studies of financial incentives for smoking cessation, but our estimates are imprecise due to lower than planned enrollment. The design of our incentive program is comparable to successful previously tested programs. For example, a study with Medicaid enrollees finds a statistically significant increase in cessation rates from a similar incentive schedule to this study: $30 per counselling call and $40 for biochemically verified smoking cessation at six months with a total incentive amount of $190 [20]. The incentives provided were large compared to the average income of study participants and so, *a priori*, expected to motivate behavior change. Relatedly, making deposit contracts a 2^nd^ stage, contingent on incentive earnings from cessation in a 1^st^ stage, dramatically increases the sample size required to identify effects of deposit contracts in settings where financial incentives have only modest 1^st^-stage effects on cessation.

Third, not all participants engaged with the website portal. For example, only 37% of treatment participants submitted a journal entry within the two-month intervention window. Internet access or literacy (which we did not measure) was likely a barrier for some, but 73% of our study population did have daily Internet access. Our intervention might be improved by better educating participants on how to use the website portal and better explaining the functioning of the deposit contracts.

We conjecture that a combination of process incentives for using formal cessation services and outcome incentives for cessation, delivered through a mix of earned incentives and deposit contracts, may be fruitful for a Medicaid population. Further exploration should focus on improving enrollment and thus increasing the statistical precision of treatment effect estimates. Future work also could test other aspects of incentive and deposit contract design and implementation. We suggest testing more frequent incentives, more opportunities to precommit incentives, more information on the likelihood of succeeding, and more information on the likelihood of reverting to smoking.

## Figures and Tables

**Table 1:** Baseline characteristics of study participants.

Characteristic	Control (*N* = 103)	Any treatment (*N* = 208)	Treatment groups			*p* value: control vs. any treatment
			Incentives only (*N* = 107)	Commitment (*N* = 42)	Precommitment (*N* = 59)	
Female	0.62	0.58	0.62	0.57	0.53	0.11
Age	45.94 (10.66)	45.25 (10.98)	45.94 (10.66)	46.17 (11.62)	43.36 (11.03)	0.05
Married	0.14	0.14	0.14	0.20	0.10	0.08
Race or ethnic group (%)						
Race: Black or African American	0.27	0.31	0.27	0.40	0.32	0.35
Race: White	0.50	0.45	0.50	0.48	0.34	0.94
Race: Other	0.22	0.24	0.22	0.12	0.34	0.39
Hispanic	0.26	0.27	0.26	0.14	0.37	0.16
Level of education (%)						
High school or lower	0.72	0.69	0.72	0.61	0.71	0.52
Associate's or Bachelor's degree	0.15	0.20	0.15	0.29	0.24	0.20
Graduate degree	0.14	0.10	0.14	0.10	0.05	0.57
Household income (%)						
$10,000 or under	0.56	0.54	0.56	0.50	0.53	0.41
$10,001 to $30,000	0.34	0.33	0.34	0.38	0.27	0.54
$30,001 to $50,000	0.03	0.06	0.03	0.12	0.08	0.79
$50,001 and above	0.07	0.07	0.07	0.00	0.12	0.39
Smoking behaviors						
Smoking more than 20 cigarettes a day	0.02	0.03	0.02	0.05	0.05	0.13
Quit attempts in the last year	2.00 (41.28)	2.00 (40.74)	2.00 (41.28)	1.00 (1.91)	1.00 (52.44)	0.79
Has quit 1 year or more since starting smoking	0.18	0.18	0.18	0.12	0.24	0.55
Fagerstrom score for nicotine dependence						
Low dependence	0.07	0.08	0.07	0.10	0.08	0.49
Low to moderate dependence	0.23	0.25	0.23	0.26	0.27	0.56
Moderate dependence	0.36	0.35	0.36	0.31	0.34	0.18
High dependence	0.17	0.15	0.17	0.19	0.10	0.67

Note: values represent means (SD) unless otherwise indicated. Median reported due to outliers (4 participants responded 365).

**Table 2:** Analysis of the treatment effect on primary and secondary outcomes.

	Incentives only		Commitment		Precommitment		Any treatment	
	OR or coef. [95% CI]	*p* value	OR or coef. [95% CI]	*p* value	OR or coef. [95% CI]	*p* value	OR or coef. [95% CI]	*p* value
No demographic controls								
Primary outcomes (OR)								
Passed urine test at 2 m	1.36 [0.52 : 3.53]	0.53	1.98 [0.64 : 6.10]	0.23	1.86 [0.66 : 5.26]	0.24	1.62 [0.70 : 3.73]	0.26
Passed urine test at 6 m	2.48 [0.47 : 13.06]	0.29	1.23 [0.11 : 13.96]	0.87	4.68 [0.88 : 24.91]	0.07	2.82 [0.61 : 12.97]	0.18
Secondary outcomes (OLS coefficients)								
Total support counselling sessions attended	0.25 [-0.66 : 1.16]	0.59	-0.21 [-0.80 : 0.38]	0.48	-0.06 [-0.82 : 0.69]	0.87	0.07 [-0.55 : 0.69]	0.82
Total support prescriptions given	-0.17 [-0.53 : 0.19]	0.35	-0.24 [-0.59 : 0.12]	0.20	-0.31 [-0.75 : 0.13]	0.17	-0.22 [-0.53 : 0.08]	0.15

With demographic controls								
Primary outcomes (OR)								
Passed urine test at 2 m	1.71 [0.53 : 5.51]	0.37	3.24 [0.86 : 12.25]	0.08	3.03 [0.85 : 10.79]	0.09	2.31 [0.81 : 6.60]	0.12
Passed urine test at 6 m	0.89 [0.13 : 6.03]	0.90	0.84 [0.06 : 11.07]	0.89	4.08 [0.70 : 23.90]	0.12	1.65 [0.33 : 8.37]	0.54
Secondary outcomes (OLS coefficients)								
Total support counselling sessions attended	0.08 [-0.74 : 0.89]	0.86	-0.16 [-0.93 : 0.61]	0.68	-0.36 [-1.14 : 0.43]	0.37	-0.09 [-0.66 : 0.48]	0.75
Total support prescriptions given	-0.16 [-0.52 : 0.21]	0.40	-0.19 [-0.58 : 0.20]	0.34	-0.33 [-0.70 : 0.04]	0.08	-0.21 -0.52 : 0.09]	0.17

Note: usual care is the reference group. Logistic regressions for the binary outcomes “passed urine test at 2 m” and “passed urine test at 6 m” and ordinary least squares (OLS) for the remaining outcomes. OR refers to the odds ratio from a logistic regression, and coef. refers to the coefficient from an OLS regression. Demographic controls include age, sex, an indicator for high school as the highest education level, and income bin indicators.

## Data Availability

The data reported in this paper have been deposited in the Innovations for Poverty Action Dataverse (https://dataverse.harvard.edu/dataverse/ipa) (doi:10.7910/DVN/T2HN28).
